# Prevalence of Molar Incisor Hypomineralization Among Children Aged Eight to 12 Years on Asthma Medication in Chengalpattu District, India: A Cross-Sectional Study

**DOI:** 10.7759/cureus.94085

**Published:** 2025-10-07

**Authors:** Pasumpon Muthupandian, Nagappan Nagappan

**Affiliations:** 1 Public Health Dentistry, Chettinad Dental College and Research Institute, Chennai, IND

**Keywords:** asthmatic medication, enamel defects, molar incisor hypomineralisation (mih), pediatric dentistry, respiratory illness and oral health

## Abstract

Background

Molar incisor hypomineralization (MIH), a defect in enamel development, is linked to early childhood illnesses. While respiratory infections correlate with increased MIH risk, the independent contribution of associated medications, particularly antibiotics, remains unclear, creating a significant knowledge gap.

Aim

The aim of the study is to investigate the prevalence of MIH in children aged eight to 12 years from Chengalpattu district, Tamil Nadu, India, who used asthmatic medication during their first three years of life.

Materials and methods

A cross-sectional study was conducted in the Chengalpattu district, examining 266 children aged eight to 12 years who had used asthmatic medication in their first three years of life. MIH was assessed using the European Archives of Pediatric Dentistry (EAPD) diagnostic criteria, and data were collected via structured questionnaires and clinical examinations at Chettinad Hospital and Research Institute. Data were analyzed using IBM SPSS Statistics software, version 24 (IBM Corp., Armonk, NY). Chi-square and Kolmogorov-Smirnov tests were employed to assess categorical data and normality, respectively, with a significance level of p < 0.05.

Results

A significant association (p < 0.001) was found between asthmatic medication use and MIH. Specifically, corticosteroid inhaler use was linked to a range of MIH clinical presentations and lesion severities, while beta-2 agonists combined with antihistamines were associated with more severe lesion extents.

Conclusion

This study concludes that a significant association exists between asthmatic medication, particularly corticosteroid inhalers and beta-2 agonist/antihistamine combinations, and MIH severity in children. Developing and implementing targeted oral health strategies, including early dental surveillance and preventive interventions, is crucial for mitigating MIH in asthmatic children.

## Introduction

Molar incisor hypomineralization (MIH) is a condition where the enamel of teeth doesn't form properly. This faulty formation can happen during any of the stages of enamel development: the presecretory stage, the secretory stage, the transition stage, the maturation stage, and the post-maturation stage [1,2]. MIH, also known as non-fluoride enamel opacities, internal enamel hypoplasia, nonendemic mottling of enamel, opaque spots, idiopathic enamel opacities, and idiopathic enamel hypomineralization, was introduced in 2001 [3,4]. MIH is a concerning dental development defect affecting children [5]. It manifests as demarcated opacities, ranging from white to yellow/brown, on the permanent first molars and occasionally the incisors. These changes exhibit a spectrum of severity, ranging from small, distinct opacities to substantial structural damage, termed post-eruptive breakdown (PEB), which affects significant portions of the tooth's crown and cusp areas [6]. Clinically, the hypomineralized enamel can be soft, porous, or resemble discolored chalk or old Dutch cheese [4,7].

The previous European Archives of Pediatric Dentistry (EAPD) policy document raised concerns that the current definition of MIH may be too restrictive, potentially leading to an underestimation of the true prevalence of this enamel defect, as similar demarcated opacities have been observed on permanent canine cusps, second permanent molars, and premolars [8].

It can sometimes present with opacities in the upper and lower incisors. The risk of defects to the upper incisors appears to increase with the increasing number of affected first permanent molars (FPMs) [9]. The defects of incisors are usually without loss of enamel substance and are generally less serious than those seen in molars due to the absence of chewing forces [3,9]. Demarcated opacities are also observed in second primary molars, termed hypomineralized second primary molars (HSPM), which have been predictive of MIH [8]. These weakened areas are prone to fractures under chewing pressure [3]. The condition poses challenges for dental care due to the altered morphology and structure of enamel prisms, leading to porosity, fragility, increased susceptibility to caries, and hypersensitivity [3, 10]. When PEB occurs, the enamel breaks down after the tooth erupts, which exposes the underlying dentin. This exposure makes the tooth more sensitive and increases the risk of cavities [3]. While the criteria for diagnosing MIH are established, the exact cause of this condition remains unclear [2].

It is believed to arise from disruptions during the enamel apposition and maturation phases. Studies suggest a multifactorial origin, involving genetic susceptibility, environmental, and systemic factors [1,11]. Health problems during pregnancy, such as diabetes, hypertension, and vitamin D deficiency, have been associated with a higher risk of MIH. Perinatal complications, including oxygenation deficiencies during birth, and early childhood illnesses, such as allergies, asthma, and respiratory infections, have also been implicated [7]. Additionally, prenatal exposure to medications like amoxicillin and corticosteroids during amelogenesis has been hypothesized to alter enamel formation, though definitive evidence is lacking [12,13].

The first permanent molar enamel formation (amelogenesis) occurs from the 28^th^ gestational week [2]. The ameloblast, the cell responsible for producing enamel, is among the most sensitive cells in the human body: if its function is temporarily or permanently interrupted, depending on the time of injury, hypomineralization of the enamel might occur. Some diseases in childhood can lead to problems in the supply of oxygen to ameloblasts, which causes a loss of the mineral-secreting capacity of these cells [14,15]. One of the main hypotheses for the emergence of MIH comes from the lack of oxygen, mainly through diseases that occurred in the perinatal period, such as asthma, bronchitis, pneumonia, and rhinitis, which may cause an imbalance of oxygen in the head and neck region.

A systematic review reported an association between MIH and respiratory diseases [2]. The primary respiratory diseases that appeared as postnatal events related to MIH were asthma, pneumonia, tonsillitis, and bronchitis. Corticosteroid therapy commonly used by asthmatic children is known to suppress osteoblast formation and activity, resulting in decreased bone formation [2,16].

The association of MIH with antibiotic use is somewhat unclear. Because antibiotics are commonly used for upper respiratory infections, it is not possible to confirm whether the association was caused by the disease or the drug [3]. The association of MIH with fever is also inconclusive. Ameloblasts are highly susceptible to relatively minor changes in their environment. Increases in temperature, hypocalcemia, and pH shifts can all disrupt the normal process of amelogenesis [16-18]. The study recruited children aged eight to 12 years so that a proper assessment of MIH could be done. At this age, most children would have had all four FPMs and the majority of incisors, but these teeth would not have been exposed to the oral environment long enough to develop dental caries [3]. By investigating the prevalence of MIH among children using asthmatic medication from the first three years of life in Chengalpattu district, Tamil Nadu, India, this study aims to contribute valuable data to the existing body of knowledge on MIH etiology. The objectives of the study are as follows: To assess the prevalence of MIH among children aged eight to 12 years who used asthmatic medications during the first three years of life and to explore the possible associations between different types of asthmatic medications and the occurrence of MIH.

## Materials and methods

A cross-sectional study was conducted to assess the prevalence of MIH among children aged eight to 12 years with a history of asthmatic medication during the first three years of life. The study was carried out among children visiting the outpatient department of pediatric unit of Chettinad Hospital And Research Institute, a tertiary care hospital in the Chengalpattu district, from January 2025 to March 2025. The study protocol adhered to the Strengthening the Reporting of Observational Studies in Epidemiology (STROBE) guidelines. Ethical clearance was obtained from the Institutional Human Ethics Committee (approval number: IHEC-CDCRI/2024/STU-0135), and written informed consent was secured from the parents of all participating children.

The sample size was calculated using G*Power software version 3.1.9.4 (Heinrich-Heine-Universität Düsseldorf, Düsseldorf, Germany) based on data from a previous study by Marina Girgis Azmy et al. [19]. Using a significance level (alpha) of 1%, a design effect of 1, and a power of 95%, the required sample size was estimated to be 266. Purposive sampling was employed to recruit participants after obtaining permission from the relevant authorities of the tertiary care hospital.

Inclusion criteria consisted of children aged eight to 12 years attending the pediatric dentistry outpatient department during the study period who had a documented history of asthmatic medication usage during the first three years of life and whose parents consented to participate. Children were excluded if they had fixed orthodontic appliances, generalized enamel hypomineralization, lacked verifiable past medical records, or had a medical history unrelated to asthma that could confound the findings.

Data collection involved a face-to-face interview with the parents using a structured, English-language questionnaire, followed by an intraoral examination for the children. The questionnaire comprised one domain covering sociodemographic variables (age, gender, education, address, and location) and medical history, including the type of asthmatic medication administered during the first three years of life. A pilot test of the questionnaire was conducted on 26 children (10% of the sample) attending the pediatric unit at Chettinad Hospital and Research Institute, Chennai, to assess feasibility, and intra-examiner reliability was evaluated by re-examining a subset of 26 children after a two-week interval. The Kappa statistic for intra-examiner agreement was 0.89, indicating excellent consistency. Inter-examiner reliability was assessed by having a second independent examiner evaluate the same subset of 26 children. The Kappa statistic for inter-examiner agreement was 0.85, reflecting strong concordance between examiners. The test-retest reliability showed strong consistency, with a Cronbach’s alpha value >0.84, and these participants were not included in the main study. 

Oral examinations were conducted to assess the presence of MIH using the short-form diagnostic criteria established by the EAPD [8]. A single calibrated examiner performed all clinical evaluations using the World Health Organization (WHO) oral health survey methodology [20], employing an artificial light source, mouth mirror, and explorer. MIH findings were recorded as categorical variables. The association between MIH (both clinical status and lesion criteria) and the type of asthmatic medication used was evaluated.

Data entry was performed using Microsoft Excel (Microsoft Corp., Redmond, WA), and statistical analyses were carried out using IBM SPSS Statistics for Windows, Version 24.0 (IBM Corp., Armonk, NY). Descriptive statistics were presented as frequency distributions of sociodemographic variables. The chi-square test was used to examine associations between MIH status and type of asthmatic medication. A p-value < 0.05 was considered statistically significant, while a p-value < 0.001 was considered highly significant.

## Results

The study included 266 participants, with 140 males (52.6%) and 126 females (47.4%). Participants were aged eight to 12 years, with the majority being nine years old (n=81, 30.5%), followed by eight years (n=65, 24.4%), 10 years (n=56, 21.1%), 11 years (n=35, 13.2%), and 12 years (n=29, 10.9%). Most participants resided in urban areas (n=164, 61.7%), while the rest were from rural areas (n=102, 38.3%) (Table 1).

**Table 1:** Sociodemographic details of the participants

Variable	Frequency (N)	Percentage (%)
Gender		
Male	140	52.6%
Female	126	47.4%
Age (years)		
8	65	24.4%
9	81	30.5%
10	56	21.1%
11	35	13.2%
12	29	10.9%
Location		
Rural	102	38.3%
Urban	164	61.7%

Regarding asthmatic medication usage, 123 participants (46.2%) used corticosteroid inhalers alone, 65 (24.4%) used a combination of corticosteroids, beta-2 agonists, and antihistamines, 42 (15.8%) used corticosteroids with antihistamines, 11 (4.1%) used corticosteroids with beta-2 agonists, and 25 (9.4%) used beta-2 agonists with antihistamines (Table 2).

**Table 2:** Distribution the study participants according to the type of asthmatic medication

Type of asthmatic medication	Frequency (N)	Percentage (%)
Beta 2 agonist + antihistamine	25	9.4%
Corticosteroid inhaler	123	46.2%
Corticosteroid inhaler + beta 2 agonist	11	4.1%
Corticosteroid inhaler + antihistamine	42	15.8%
Corticosteroid inhaler + beta 2 agonist + antihistamine	65	24.4%
Total	266	100%

In terms of MIH clinical status, 140 participants (52.6%) had no visible defects. Enamel defects were observed in 41 participants (15.4%), demarcated opacities in 40 (15.0%), atypical restorations in 24 (9.0%), and post-eruptive enamel breakdown in 21 (7.9%) (Table 3).

**Table 3:** Distribution of molar incisor hypomineralization: clinical status criteria

Clinical status criteria	Frequency (N)	Percentage (%)
Atypical restoration	24	9.0%
Demarcated opacities	40	15.0%
Enamel defect	41	15.4%
No visible defect	140	52.6%
Post-eruptive enamel breakdown	21	7.9%
Total	266	100%

Analysis of MIH lesion criteria revealed that 181 participants (68.0%) had no MIH. Lesions affecting less than one-third of the tooth surface were found in 75 participants (28.2%). At least one-third but less than two-thirds of the tooth was affected in four participants (1.5%), and at least two-thirds of the tooth was affected in six participants (2.3%) (Table 4).

**Table 4:** Distribution of molar incisor hypomineralization (MIH): lesion criteria

Lesion criteria	Frequency (N)	Percentage (%)
No MIH	181	68.0%
Less than one-third of the tooth is affected	75	28.2%
At least one third but less than two-thirds	4	1.5%
At least two-thirds of the tooth is affected	6	2.3%
Total	266	100%

A statistically significant association was observed between the type of asthmatic medication and the clinical status of MIH (χ² = 43.892, df = 16, p = 0.001), with a small to moderate effect size (Cramér’s V = 0.203) (Table 5).

**Table 5:** Association between asthmatic medication and molar incisor hypomineralization (MIH): clinical status criteria Chi square test; *p < 0.05 - statistically significant

	MIH clinical status criteria										
Type of asthmatic medication		Atypical restoration	Demarcated Opacities	Enamel defect	No visible defect	Post-eruptive enamel breakdown	Total	χ²	df	V	p-value
	Beta 2 agonist + antihistamine	0	7	2	10	6	25	43.892	16	0.203	0.001*
	Corticosteroid inhaler	14	13	21	63	12	123				
	Corticosteroid inhaler + beta 2 agonist	0	0	0	11	0	11				
	Corticosteroid inhaler + antihistamine	1	4	7	29	1	42				
	Corticosteroid inhaler + beta 2 agonist+ antihistamine	9	16	11	27	2	65				
Total		24	40	41	140	21	266				

A statistically significant association was found between the type of asthmatic medication and the extent of MIH lesions (χ² = 42.000, df = 12, p = 0.001), with a small to moderate effect size (Cramér’s V = 0.229) (Table 6).

**Table 6:** Association between asthmatic medication and molar incisor hypomineralization (MIH): lesion criteria Chi square test; *p < 0.05 - statistically significant

	MIH lesion criteria									
Type of asthmatic medication		At least one third but less than two-thirds	At least two-thirds of the tooth is affected	Less than one-third of the tooth is affected	No MIH	Total	χ²	df	V	p-value
	Beta 2 agonist + antihistamine	1	4	8	12	25	42.00	12	0.229	0.001*
	Corticosteroid inhaler	1	2	36	84	123				
	Corticosteroid inhaler + beta 2 agonist	0	0	0	11	11				
	Corticosteroid inhaler + antihistamine	1	0	5	36	42				
	Corticosteroid inhaler + beta 2 agonist+ antihistamine	1	0	26	38	65				
TOTAL		4	6	75	181	266				

A highly significant association was found between clinical status and the extent of MIH lesions (χ² = 318.514, df = 12, p = 0.001), with a large effect size (Cramér’s V = 0.632) (Table 7).

**Table 7:** Association between clinical status criteria and lesion criteria for molar incisor hypomineralization (MIH) Chi square test; *p < 0.05 - statistically significant

	Lesion criteria									
Clinical status criteria		At least one third but less than two-thirds	At least two-thirds of the tooth is affected	Less than one-third of the tooth is affected	No MIH	Total	χ²	df	V	p-value
	Atypical restoration	2	1	21	0	24	318.514	12	0.632	0.001*
	Demarcated opacities	0	0	40	0	40				
	Enamel defect	0	0	0	41	41				
	No visible defect	0	0	0	140	140				
	Post-eruptive enamel breakdown	2	5	14	0	21				
TOTAL		4	6	75	181	266				

## Discussion

It is important to have an early warning of MIH since specific teeth typically show post-eruptive enamel loss, which can cause rampant advancement of caries, resulting in pain. Hence, this study assessed the prevalence of MIH in children from Chengalpattu district who were under asthmatic medication, because to the best of our knowledge, there is no data on the prevalence from this part of the world.

Building upon the foundational work of a previous study that established the prevalence of MIH in children from Chengalpattu aged eight to 12 years at 10.9%, our current research sought to delve deeper into the etiological factors contributing to this condition [21]. While that study provided valuable insights into the regional prevalence and associations with factors like body mass index (BMI), dental caries, and oral hygiene, our investigation specifically focused on the potential role of asthmatic medications [21]. Recognizing the limitations in understanding the nuanced causes of MIH, the current study aimed to explore whether asthmatic medication use, a factor not previously examined in this region, could be a significant contributor. This study has revealed a statistically significant association between asthmatic medication and MIH, expanding upon the earlier prevalence data by providing new etiological insights.

Furthermore, an advanced understanding of MIH is made in the present research by describing the specific clinical presentations and lesion criteria related to different asthmatic medications. While the prior study reported no gender predilection, the current study showed a slight male predominance. The specific clinical features of MIH were explored, and it was shown that post-eruptive breakdown was associated with specific drug combinations.

In our current study, we observed a statistically significant association between asthmatic medication use and MIH, with a notable prevalence of MIH among participants using corticosteroid inhalers, either alone or in combination with other medications. This aligns with previous research suggesting a link between corticosteroids and ameloblast activity disruption [22].

Data regarding the age at which participants initiated asthmatic medications were collected from their medical records. Thus, the current study, in part, aimed to examine the relationship between the age of medication initiation and MIH. This aspect of our research aligns with a previous study by Mastora et al. (2017), which found a weak, statistically insignificant correlation between the age of starting asthmatic drugs and MIH [23]. While we collected this data, our analysis did not specifically focus on the age of medication initiation as a distinct variable in relation to MIH.

Our finding that all participants with MIH were taking corticosteroids, either alone or in combination with beta-2 agonists and/or antihistamines, reinforces the potential role of corticosteroids in MIH development. The observed high prevalence of MIH in the corticosteroid and beta-2 agonist combination group is particularly noteworthy. As suggested by Serna et al. (2016) in their systematic review, the association between asthma medications and enamel defects is well-documented [24].

This concept underscores the importance of considering systemic factors that can disrupt dental development, particularly during the critical period of amelogenesis. Regarding the relationship between asthma and enamel defects, a significant body of research, with 82% of studies supporting an association, underscores the potential link between these conditions [25-27].

Our study reinforces the potential role of asthmatic medications, particularly corticosteroids, in MIH development. While previous research has documented the impact of glucocorticoids on bone formation, suggesting a similar effect on ameloblasts, the direct relationship between asthma drugs and enamel defects is not universally supported. In this research, it has been observed that the highest level of MIH occurred in those taking corticosteroid inhalers, either alone or in combination with other drugs. This suggests that the medication itself could play a role. However, it is important to acknowledge that some studies suggest that asthma disease could exert a higher impact on ameloblasts than the medication itself, due to hypoxia [28].

Intriguingly, the cohort study by Flexender et al. (2009) revealed that higher MIH values were observed in asthmatics not using metered-dose inhalers (MDIs), suggesting that poor asthma control and subsequent oxygen deprivation may be a more significant factor than medication use alone [29]. This is suggestive of our study finding that the method of controlling the disease also matters, and that high use of inhalers may be associated with a reduced severity of MIH. However, it is essential to emphasize that our study still found a statistically significant association and prevalence of MIH among children using inhaled asthma medications.

This apparent paradox, where inhaler use might mitigate some aspects of MIH while not entirely preventing it, paves the way for research pertaining to drug invention that could effectively control asthma and simultaneously prevent the occurrence of MIH. It highlights the need to explore novel therapeutic strategies that minimize the potential impact on amelogenesis while maintaining effective asthma management. This could involve investigating alternative drug delivery systems, developing medications with fewer enamel-disrupting side effects, or exploring preventative dental interventions tailored to asthmatic children. Furthermore, this study is notable for being the first to specifically examine and report on the association between clinical manifestations of MIH and the extent of the observed lesions.

Selection bias may have occurred due to the recruitment process (purposive sampling) and the age of medication initiation. Potential environmental and confounding factors, such as diet, fluoride exposure, and genetic predisposition, were not controlled or detailed in this study, which may limit the reproducibility of results in different settings. Future longitudinal studies with control groups and detailed clinical histories are recommended to better understand the relationship between asthma medications and MIH.

## Conclusions

This study found a significant association between asthmatic medication use, particularly corticosteroid inhalers and their combinations, and MIH in children aged eight to 12 years. These findings highlight the need for integrated care approaches where dental and medical professionals collaborate to monitor oral health in asthmatic children. Early dental interventions and awareness among caregivers can help mitigate long-term oral complications. 
